# Intraoperative voltage mapping-assisted resection of a giant right ventricular giant tumor: a case report

**DOI:** 10.1186/s44215-025-00239-w

**Published:** 2026-01-16

**Authors:** Takehiro Nakajima, Shun-Ichiro Sakamoto, Ryo Maekawa, Anna Tsuji, Motohiro Maeda, Kenji Suzuki, Jiro Honda, Norio Motoda, Tetsuro Sekine, Yosuke Ishii

**Affiliations:** 1 Department of Cardiovascular Surgery, Nippon Medical School Musashikosugi Hospital, 1-383 Kosugimachi, Nakahara-ku, Kawasaki-shi, Kanagawa 211-8533 Japan; 2 Department of Cardiovascular Surgery, Nakagami Hospital, Okinawa, Japan; 3 Department of Diagnostic Pathology, Nippon Medical School Musashikosugi Hospital, Kanagawa, Japan; 4 Department of Radiology, Nippon Medical School Musashikosugi Hospital, Kanagawa, Japan; 5 Department of Cardiovascular Surgery, Nippon Medical School, Tokyo, Japan

**Keywords:** Cardiac tumor, Hemangioma, Cardiac mapping

## Abstract

**Background:**

Cardiac hemangiomas located in the right heart can lead to serious complications, including right ventricular outflow tract (RVOT) obstruction, arrhythmias, and sudden cardiac death. Although surgical resection remains the primary treatment, complete excision of intramural tumors poses risk of impairing cardiac function. This case report describes the successful resection of a large right ventricular hemangioma using intraoperative voltage mapping, which enable maximal tumor removal while preserving myocardial integrity and preventing postoperative heart failure.

**Case presentation:**

A 76-year-old female underwent a routine health examination in July 2024, during which cardiomegaly and nonspecific ST-segment changes were detected on electrocardiography. Contrast enhanced computed tomography revealed a well-defined 60 mm mass within the right ventricle, causing significant ROVT stenosis. No evidence of distant metastasis or elevated tumor markers was detected. The patient underwent tumor resection via median sternotomy. Intraoperative voltage mapping was utilized to delineate viable myocardium at the tumor margins. The tumor was excised while preserving functional myocardial tissue. Cryoablation was performed at the resection margins, and resultant defect in the right ventricular wall was reconstructed using a bovine pericardial patch. Histopathological analysis confirmed the diagnosis of cardiac hemangioma. The patient experienced an uneventful postoperative course had no postoperative complications and was discharged on postoperative day 16. Preoperative and postoperative cardiac magnetic resonance imaging demonstrated preserved right ventricular function.

**Conclusions:**

Intraoperative voltage mapping proved to be a valuable adjunct in the surgical management of right ventricular tumors, enabling effective tumor resection while preserving myocardial tissue and maintaining postoperative cardiac function.

**Supplementary Information:**

The online version contains supplementary material available at 10.1186/s44215-025-00239-w.

## Backgrounds

Primary cardiac tumors are exceedingly rare, with an incidence of only 0.05% − 0.2% in autopsy series. Among these, cardiac hemangiomas present an exceptionally uncommon subset, accounting for just 2% − 2.8% of all primary cardiac tumors [1, 2]. Although typically asymptomatic, cardiac hemangiomas may present with a range of clinical manifestations depending on their anatomic location. Tumors arising in the right heart are more frequently observed and are particularly associated with right ventricular outflow tract (RVOT) obstruction, conduction system disturbances, and ventricular arrhythmias, potentially leading to syncope, stroke, or sudden cardiac death [3, 4].

Surgical resection remains the mainstay of the treatment; however, complete excision of intramural hemangiomas is often complicated by the need to preserve myocardial contractility. Here, we present the case of a large intramural hemangioma located in the RVOT, which was successfully and completely resected with the aid of intraoperative voltage mapping. This technique facilitated myocardial preservation and resulted in excellent postoperative right ventricular function without signs of heart failure. Written informed consent for publication of the patient’s clinical details and images was obtained before manuscript preparation.

## Case presentation

A 76-year-old female was referred to our hospital in July 2024 after cardiomegaly was incidentally detected on chest radiography and nonspecific ST-segment changes were observed on electrocardiography during a routine medical checkup. Transthoracic echocardiography performed at a local clinic revealed a 60-mm mass within the right ventricle. The patient was subsequently referred to our hospital for further evaluation.

**Fig. 1:**
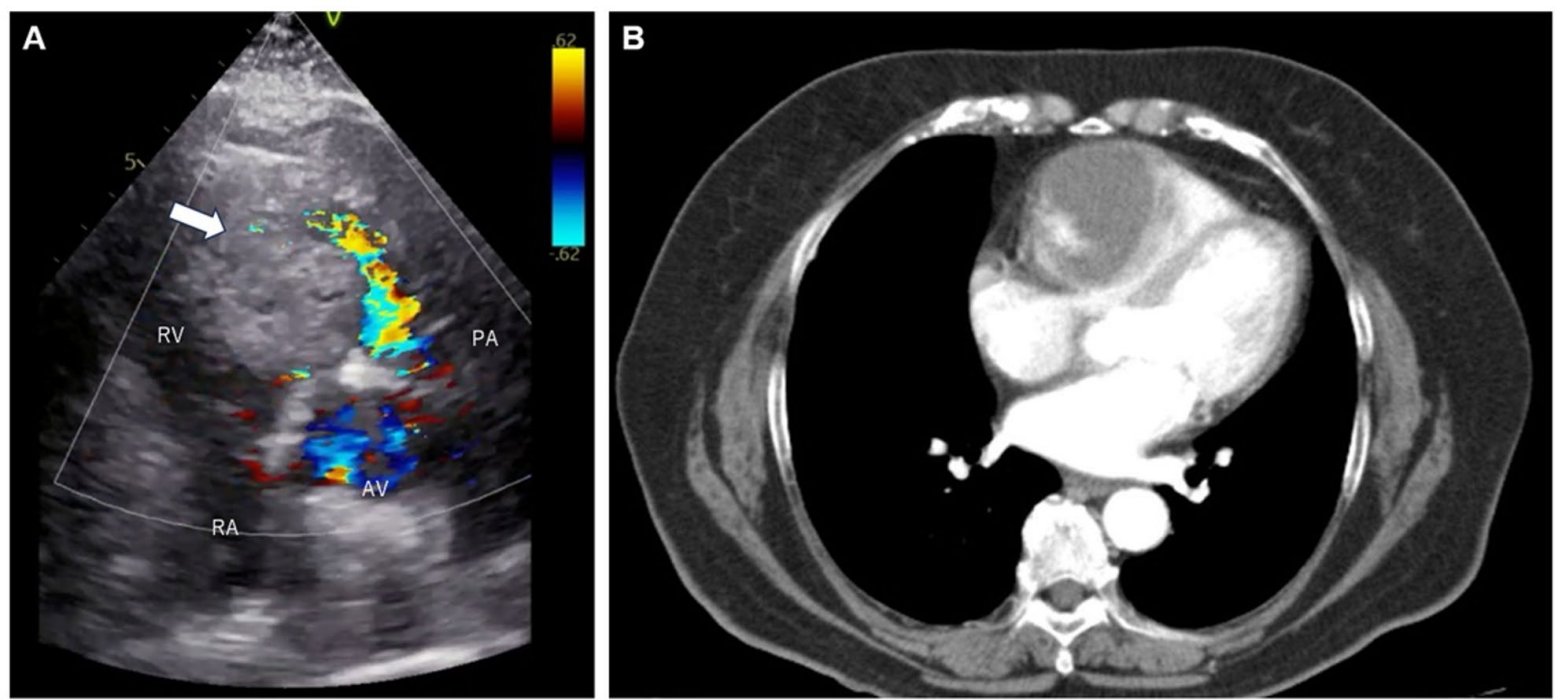
Preoperative imaging findings. (**a**) Transthoracic echocardiography revealed a right ventricular mass approximately 6 cm in diameter (white arrow), with mosaic flow indicating right ventricular outflow tract stenosis. (**b**) Contrast-enhanced CT demonstrated a heterogeneously enhancing mass, with contrast uptake extending from the periphery toward the center. RV, right ventricle; RA, right atrium; AV, aortic valve; PA, pulmonary artery

Serum tumor marker levels were within normal limits. Repeat transthoracic echocardiography at our institution confirmed a right ventricular mass measuring approximately 6 cm in diameter, accompanied by mosaic flow signals around the tumor and evidence of RVOT stenosis. with right ventricular outflow tract stenosis (Fig. 1a). Contrast-enhanced computed tomography (CT) revealed a 61 mm×42 mm heterogeneously enhancing mass in the right ventricle, with no apparent connection to the coronary arteries (Figure.1b). Cardiac magnetic resonance imaging (MRI) demonstrated a poorly demarcated intramural tumor within the right ventricular myocardium. The boundary between the tumor and adjacent myocardium was indistinct, and the endocardial myocardium appeared thinned, raising concerned about myocardial invasion. No distant metastases were identified.

Based on the imaging findings, differential diagnosis included cardiac hemangioma and hemangiosarcoma. Given the tumor’s size and location, surgical resection was deemed technically feasible. A multidisciplinary team—including specialists from anesthesiology, cardiovascular surgery, and radiology—reviewed the case, and surgery was planned with the assistance of intraoperative voltage mapping to guide resection.

**Fig. 2:**
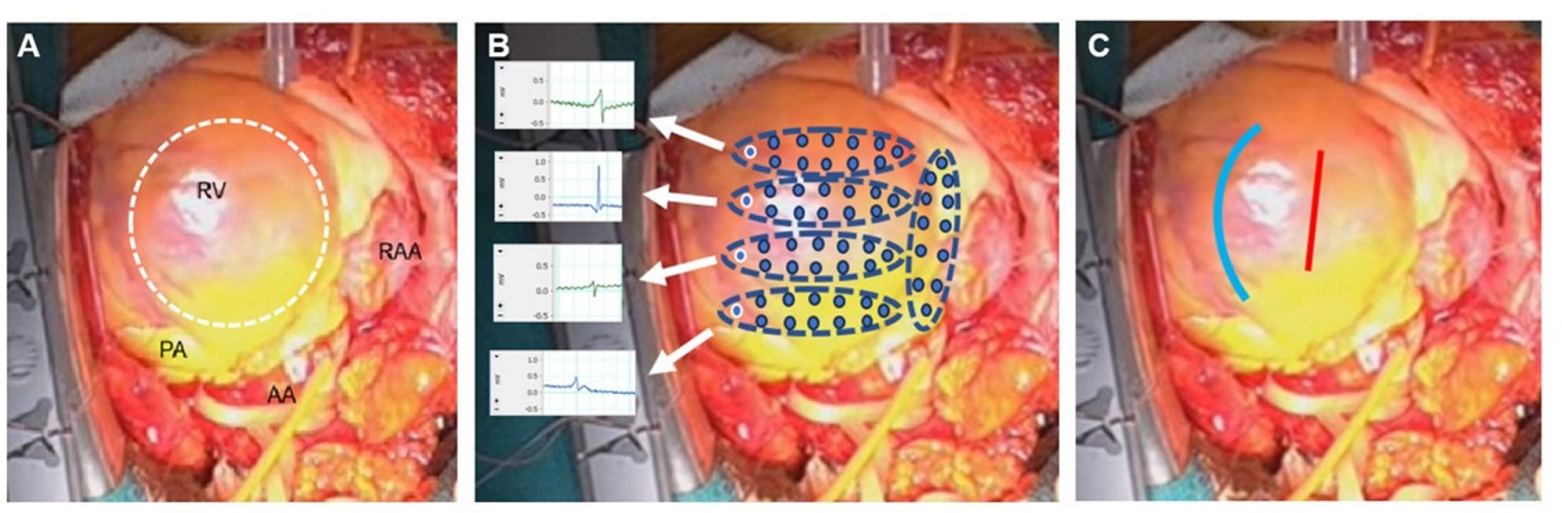
Intraoperative voltage mapping. (**a**) The tumor, covered by epicardium, appeared to bulge from the right ventricular free wall toward the outflow tract (outlined by white dotted circle). (**b**) Cardiac voltage mapping of the right ventricular free wall was performed using oval electrode patches, each containing 12 bipolar electrodes placed at five distinct locations. Electrical potentials were detectable at only four positions. Blue circles indicate bipolar electrodes; dotted circles, electrode patches; and white circles, sites where potentials were recorded (dotted arrows). (**c**) The blue line demarcates the myocardial preservation margin, and the red line indicates the planned site for right ventriculostomy. RV, right ventricle; RAA, right atrial appendage; PA, pulmonary artery; AA, Ascending Aorta

**Fig. 3:**
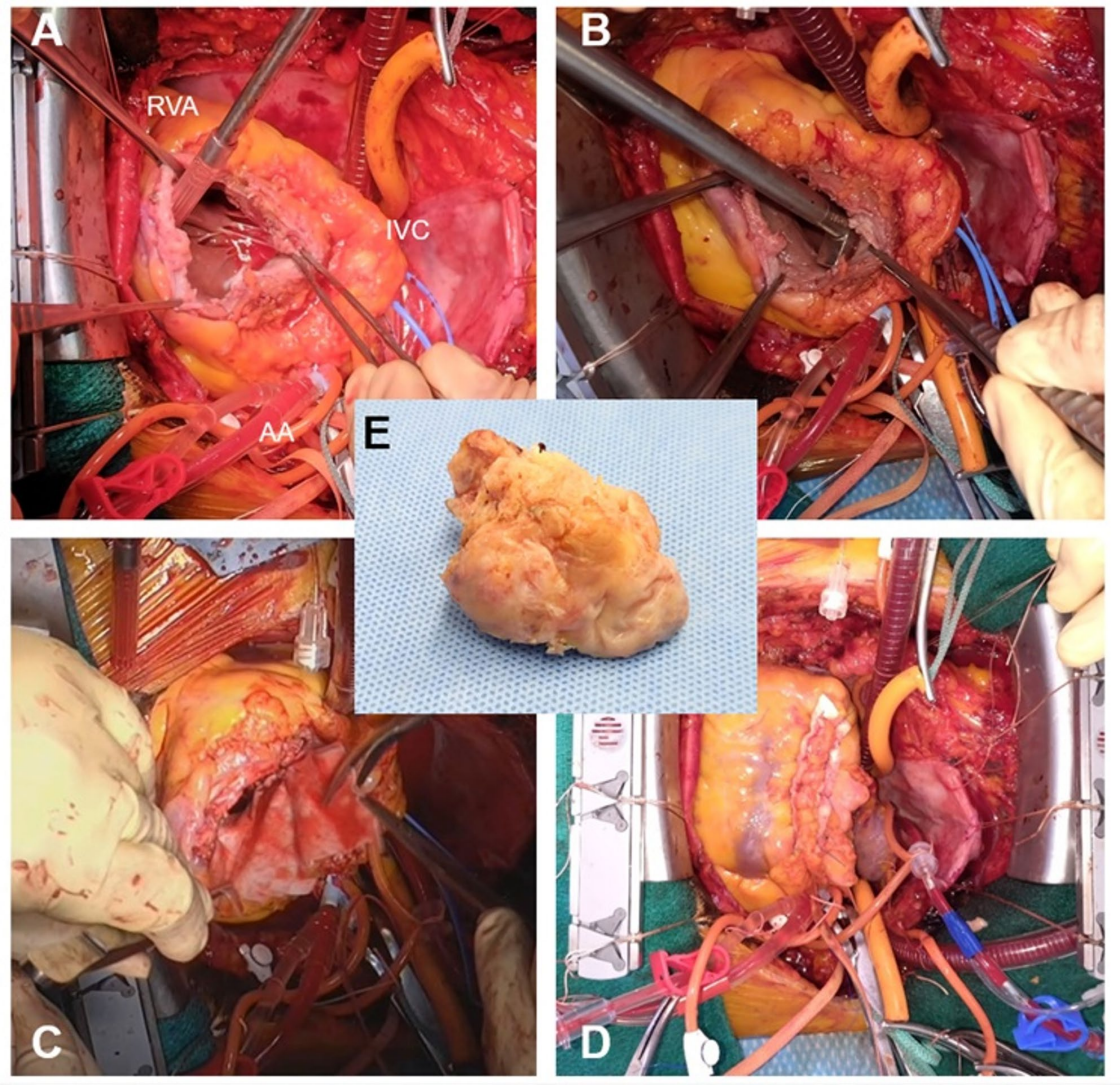
Intraoperative findings and excised specimens. (**a**) The papillary muscles, chordae tendineae, and tricuspid valve were preserved after tumor excision. (**b**) Circumferential cryoablation was performed at the resection margin. (**c**) Right ventriculoplasty was conducted by patching the endocardial defect with bovine pericardium. (**d**) Closure of the epicardial surface was achieved using two felt strips. (**e**) The tumor was resected in multiple fragments, with the largest specimen measuring 5 × 3 × 2 cm. RVA, right ventricular apex; IVC, inferior vena cava; AA, Ascending aorta

Surgical access was obtained via median sternotomy. Intraoperative voltage mapping of the right ventricular free wall was performed using a custom-designed oval electrode patch equipped with 12 bipolar electrodes positioned at five predefined locations. Bipolar electrograms were recorded with a gain setting of 250. A four-channel custom-built mapping system displayed digitized bipolar signals with dial switching. Intraoperative images of the heart were superimposed with electrode positioning data to accurately identify residual viable myocardium (Fig. 2a, b). After establishing cardiopulmonary bypass with femoral arterial cannulation and bicaval venous drainage, the aorta was cross-clamped. Cardiac arrest was achieved by administration of bidirectional cardioplegia. A longitudinal incision was made midway between the preserved myocardium and the interventricular groove (Fig. 2c). Upon opening the thickened tumor capsule, dark-red hemorrhagic fluid was released, and a substantial intramural mass was exposed. The macroscopic boundary between the tumor and the normal myocardium was relatively well defined. Tumor resection was performed primarily along the endocardial surface, with special attention to preserving areas of viable myocardium identified by voltage mapping. After complete excision, cryoablation was applied to the resection margins to reduce the risk of recurrence. To prevent undue tension on the right ventricular endocardial defect, a bovine pericardial patch (7 × 3 cm) was applied for right ventricular repair, followed by epicardial closure reinforced with two felt strips. (Fig. 3a-e).

**Fig. 4:**
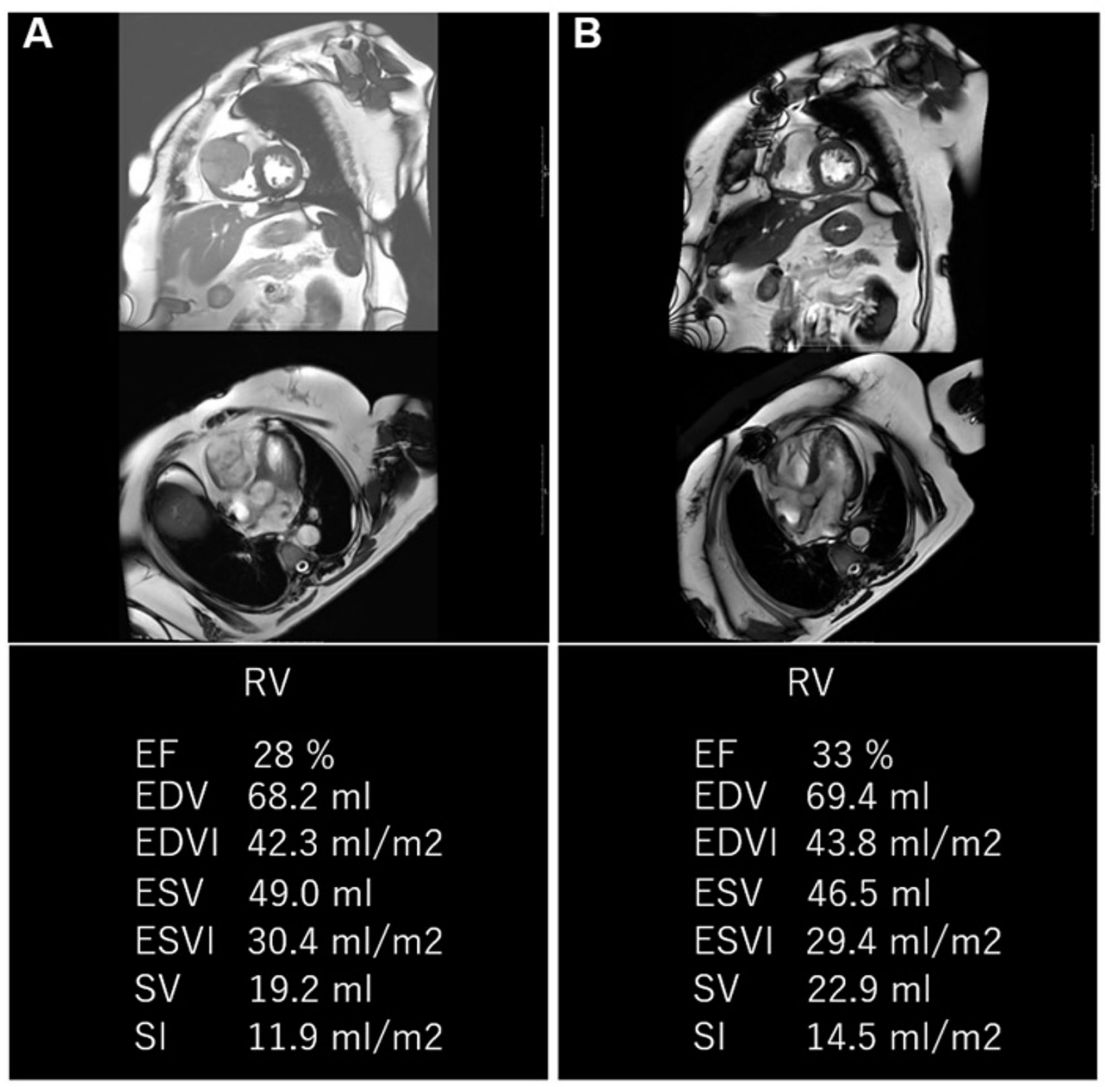
Preoperative and postoperative assessment of right ventricular (RV) size and function using cardiac magnetic resonance imaging (MRI). (**a**, **b**) Sagittal and horizontal views of the right and left ventricles obtained before and after surgery, respectively. The lower panels show quantitative parameters of RV function. Postoperative evaluation indicated mild improvement in both end-diastolic volume and contractility. EF, ejection fraction; EDV, end-diastolic volume; EDVI, end-diastolic volume index; ESV, end-systolic volume; ESVI, end-systolic volume index; SV, stroke volume; SVI, stroke index

The total operative time was 269 min. The patient was extubated on postoperative day 1 and exhibited no signs of right heart failure. She was transferred to the general ward on postoperative day 4. Histopathological examination confirmed the diagnosis of a cardiac hemangioma. The postoperative course was uneventful, and the patient was discharged on postoperative day 16. Follow-up echocardiography demonstrated resolution of RVOT obstruction. Postoperative cardiac MRI six months postoperatively demonstrated no significant change in right ventricular contractility, end-diastolic volume, or stroke volume compared with preoperative measurements. Quantitative analysis showed comparable values for right ventricular ejection fraction (preoperative 28%, postoperative 33%), end-diastolic volume (preoperative 68.2 mL, postoperative 69.4 mL), and stroke volume (preoperative 19.2 mL, postoperative 22.9 mL), indicating preservation of right ventricular systolic function (Fig. 4). At the one-year follow-up, the patient remained asymptomatic, with no clinical evidence of tumor recurrence.

## Discussion

Cardiac hemangiomas are histologically benign but clinically significant tumors. Despite their benign pathology, no standardized treatment guidelines exist for cardiac tumors. Surgical resection is generally pursued for all primary cardiac tumors, regardless of histology, due to the risk of severe complications such as obstruction, arrhythmias, and sudden cardiac death. The postoperative prognosis varies by tumor type; notably, cardiac hemangiomas have a comparatively poorer prognosis than other benign cardiac tumors [5]. While partial resection aiming at volume reduction has been reported to relieve symptoms, particularly arrhythmias, in cardiac fibromas [6], partial resection of cardiac hemangiomas has generally been associated with unfavorable outcomes, including recurrence and conduction disturbances. This has been demonstrated in a large-scale study from a high-volume Chinese center, which comprehensively analyzed 200 surgical cases [3].

Advanced imaging modalities are indispensable not only for preoperative diagnosis but also for surgical planning and prognostication [7]. In the present case, contrast-enhanced CT revealed relatively well-demarcated tumor margins, whereas magnetic resonance imaging showed signs suggestive of intramural hemorrhage and myocardial invasion. These findings made it difficult to distinguish the lesion from a malignant primary tumor such as hemangiosarcoma [8]. After multidisciplinary consultation, we opted for curative surgery without prior biopsy, although concerns remained regarding potential impairment of right ventricular function depending on the extent of resection.

In malignant tumors, complete excision with negative surgical margins is standard, and extensive resection may necessitate postoperative mechanical circulatory support [9]. However, prior studies suggest that even after resection of up to 30% of the right ventricular myocardium, postoperative cardiac function can be adequately preserved [10]. In this case, we used intraoperative voltage mapping to identify and preserve viable myocardium surrounding the tumor, enabling more precise surgical planning to minimize functional loss.

Intraoperative cardiac mapping has traditionally been employed in arrhythmia surgery, particularly for atrial fibrillation. It has facilitated targeted approaches such as pulmonary vein isolation or left atrial incision, avoiding more invasive procedures like the Cox-Maze surgery that require biventriculotomy and extensive ablation [11]. Unlike activation mapping, which identifies conduction pathways, the voltage mapping technique used in this case delineated regions of viable and nonviable myocardium based on the amplitude of electrical potentials. We have previously reported a case of cardiac tumor surgery performed in collaboration with cardiologists using a 3D electro-anatomical mapping system (CARTO). Although a custom-made mapping system was used in the present case, the CARTO system is widely available and can be utilized in other institutions [12]. Marchlinski et al. [13] proposed that a bipolar voltage < 1.5 mV indicates low-voltage myocardium, while < 0.5 mV reflects scar tissue, based on validation using a bipolar catheter electrode. In our study, however, resection lines were planned based on the presence or absence of detectable potentials, rather than absolute voltage thresholds. While some intramural tumors may retain electrical activity on their surfaces [12], there have also been reports that tumor-infiltrated marginal tissue can exhibit local conduction delay and even form reentry circuits associated with ventricular arrhythmias [14]. However, no arrhythmias were observed in the present case. Epicardial fat and stretched myocardium may have attenuated the signal amplitude, influencing the bipolar recordings. Thus, interpretation of voltage mapping data in the context of intramural tumors requires case-specific judgment. Although the present case involved a benign tumor, if it were malignant, the possibility of infiltration should be considered, and resection of the myocardial tissue showing voltage activity on the tumor surface might be necessary. Furthermore, several reports have described determining the treatment strategy based on preoperative or intraoperative biopsy findings [15, 16]. In situations such as the present case, where imaging does not clearly define the borders of an intramural lesion, performing a biopsy may therefore be helpful in guiding surgical planning. Our findings suggest that intraoperative voltage mapping is a valuable adjunct in the surgical management of benign cardiac tumor of right ventricle, enabling maximal tumor excision while preserving surrounding viable myocardium. This approach may contribute to the maintenance of postoperative right ventricular function and should be considered in similar cases.

## Conclusions

The use of intraoperative voltage mapping during surgical resection of right ventricular tumors proved valuable in identifying and preserving viable myocardium. This technique enabled complete tumor excision while minimizing damage to the surrounding cardiac tissue, thereby preserving postoperative right ventricular function. Voltage mapping may serve as an effective adjunct in the surgical management of complex intramural cardiac tumors, particularly when functional preservation is a priority.

## Supplementary Information

Supplementary Material 1.

## Data Availability

not applicable.

## Abbreviations

RVOT: Right ventricular outflow tract
CT: Computed tomography
MRI: Magnetic resonance imaging
