# Supplementary material for: Intraoperative voltage mapping-assisted resection of a giant right ventricular giant tumor: a case report
Source: Gen Thorac Cardiovasc Surg Cases. 2026 Jan 16;5:4. doi: 10.1186/s44215-025-00239-w (PMC12896158; doi:10.1186/s44215-025-00239-w)
Supplement: Supplementary file 1 — Supplementary Material 1. [file 44215_2025_239_MOESM1_ESM.docx]

Nov 8^th^ 2025

Tetsuro Uchida

Editor

*General Thoracic and Cardiovascular Surgery Cases*

Dear Editor:

Please find enclosed our manuscript entitled “Intraoperative Voltage Mapping–Assisted Resection of a Giant Right Ventricular Tumor: A Case Report,” which we request you to consider for publication as a *Case Report* in *General Thoracic and Cardiovascular Surgery Cases*.

We sincerely appreciate your time and effort in reviewing our case report.
In accordance with the comments provided by the two reviewers, we have carefully revised the manuscript.
A detailed response to each comment has been prepared, and all corresponding revisions have been clearly indicated in the revised manuscript. We would be grateful if you could kindly consider our revised submission for re-evaluation.

This manuscript has not been published elsewhere and is not under consideration by another journal. We have approved the manuscript and agree with submission to *General Thoracic and Cardiovascular Surgery Cases*. There are no conflicts of interest to declare.

We look forward to hearing from you at your earliest convenience.

Sincerely,

Shun-Ichiro Sakamoto

Department of Cardiovascular Surgery, Nippon Medical School Musashikosugi Hospital

1-383 Kosugimachi, Nakahara-ku, Kawasaki, Kanagawa

211-8533 JAPAN

Phone No:81-44-733-5181

Email Address:saka-165@nms.ac.jp
